# Research on the online service mechanism of internet hospital in infectious disease prevention and control

**DOI:** 10.3389/ebm.2025.10349

**Published:** 2025-04-25

**Authors:** Xin Zhao, Haitao Huang, Guojun Zeng, Qingke Shi, Peijia Zhu, Longhao Zhang, Lei Li, Lunxu Liu, Nan Huang, Wenguang Liu, Kexin Yu

**Affiliations:** ^1^ Emergency Office, West China Hospital, Sichuan University, Chengdu, China; ^2^ Department of Computer Application, Chengdu College of University of Electronic Science and Technology of China, Chengdu, China; ^3^ Division of Vascular Surgery, Department of General Surgery, West China Hospital, Sichuan University, Chengdu, China; ^4^ Department of Vascular Surgery, The People’s Hospital of Leshan, Leshan, China; ^5^ Information Center, West China Hospital, Sichuan University, Chengdu, China; ^6^ Department of Science and Technology, West China Hospital, Sichuan University, Chengdu, China; ^7^ Double First-Class Construction Office, West China Hospital, Sichuan University, Chengdu, China; ^8^ Department of Clinical Research Management, West China Hospital, Sichuan University, Chengdu, China; ^9^ Department of Thoracic Surgery, and President’s Office, West China Hospital, Sichuan University, Chengdu, China; ^10^ Department of Dermatology and Venerology, West China Hospital, Sichuan University, Chengdu, China

**Keywords:** internet hospital, online service, infectious disease, prevention, control internet hospital

## Abstract

Infectious diseases can sometimes lead to pandemics, often transmitted through public and social gatherings, including in-person hospital visits. Consequently, there is an urgent need for innovative approaches to prevent their spread. Taking COVID-19 as an example, we have explored a remote, contactless hospital online model that offers the public online medical consultations, professional psychological counseling, and chronic disease management consultations, thereby mitigating the risk of new transmissions resulting from hospital visits. This model was implemented, validated, and practiced at West China Hospital in China from 29 January 2020, to 12 March 2020. It was also applicable to other infectious diseases, such as influenza A. In this research, we utilized the hospital’s internet platform, supplemented by telephone services, to offer the following to the public: 1) General medical education and consultation related to epidemics and psychological anxiety; 2) Online screening for at-risk populations; 3) Online prescription and medication delivery services for patients with chronic diseases. Consequently, over a period of more than 1 month, the online epidemic platform completed a total of 32,755 cases, including 8,783 internet consultations and 1,082 telephone consultations for the public, as well as 22,890 internet consultations for chronic disease patients. Among these, 289 high-risk individuals were identified, with 3 cases confirmed as COVID-19 during follow-up diagnoses, while no infections were detected in the remaining individuals. In conclusion, this innovative medical model serves as a significant supplement to existing healthcare systems and has the potential to be expanded to other hospitals and other infectious diseases. It is particularly beneficial in scenarios where medical resources are limited, populations are under quarantine, and there is a large demand for medical services and anxiety management during infectious disease pandemics.

## Impact statement

To the best of our knowledge, this is the first online hospital strategy and implementation scheme executed in China during an infectious disease pandemic. This strategy leveraged the pre-established infrastructure of the hospital’s internet hospital, integrating clinicians, pharmacies, IT engineers, and logistics companies to serve a population of approximately 15 million people in the surrounding areas. Besides, the online clinical screening and prescription services for chronic disease patients significantly reduced the human-to-human transmission of infectious diseases, while also alleviating psychological stress for both clinicians and the public. The experience of chronic disease patients in seeking medical care became more convenient than before. Overall, since this is a novel clinical model, there is a greater need for the public to have more understanding and trust in this system, with a view to better application during major infectious disease outbreaks.

## Introduction

Infectious diseases can sometimes pose global challenges, as exemplified by the novel coronavirus pneumonia (NCP) pathogen (COVID-19) [1, 2]. Symptoms include fever, fatigue, and dry cough, with additional symptoms such as difficulty breathing appearing later [3–5]. Different variants may present with different symptoms. At times, an epidemic can escalate rapidly into an outbreak, becoming a global public health challenge [6]. In epidemic prevention and control, it is crucial to identify the source of transmission, reduce population movement, and expedite the screening of at-risk individuals. Some individuals contracted the virus during medical visits, and healthcare workers were infected while interacting with undiagnosed or diagnosed patients [7, 8]. More concerning is that infections can also occur between asymptomatic contacts. A non-contact, remote online consultation model in hospitals can reduce population movement and lower the risk of human-to-human transmission, making it a viable approach to achieving this goal.

Our hospital’s internet hospital began operations at the end of 2018, providing a technical platform to support online consultations for infectious diseases. In the event of an infectious disease outbreak, we facilitated prevention and control efforts through online consultations via the internet hospital. The public received medical advice related to diseases and professional psychological counseling for anxiety, fear, and paranoia induced by infectious diseases through online and telephone consultations. High-risk individuals were screened, and the needs of chronic disease patients for regular medical visits were met. The number of patients visiting the hospital decreased, reducing the potential for cross-infection. Under the circumstances, panic and despair were alleviated possibly. This study was approved by the hospital’s ethics committee.

## Materials and methods

### Overall design of the hospital’s online strategy

**FIGURE 1:**
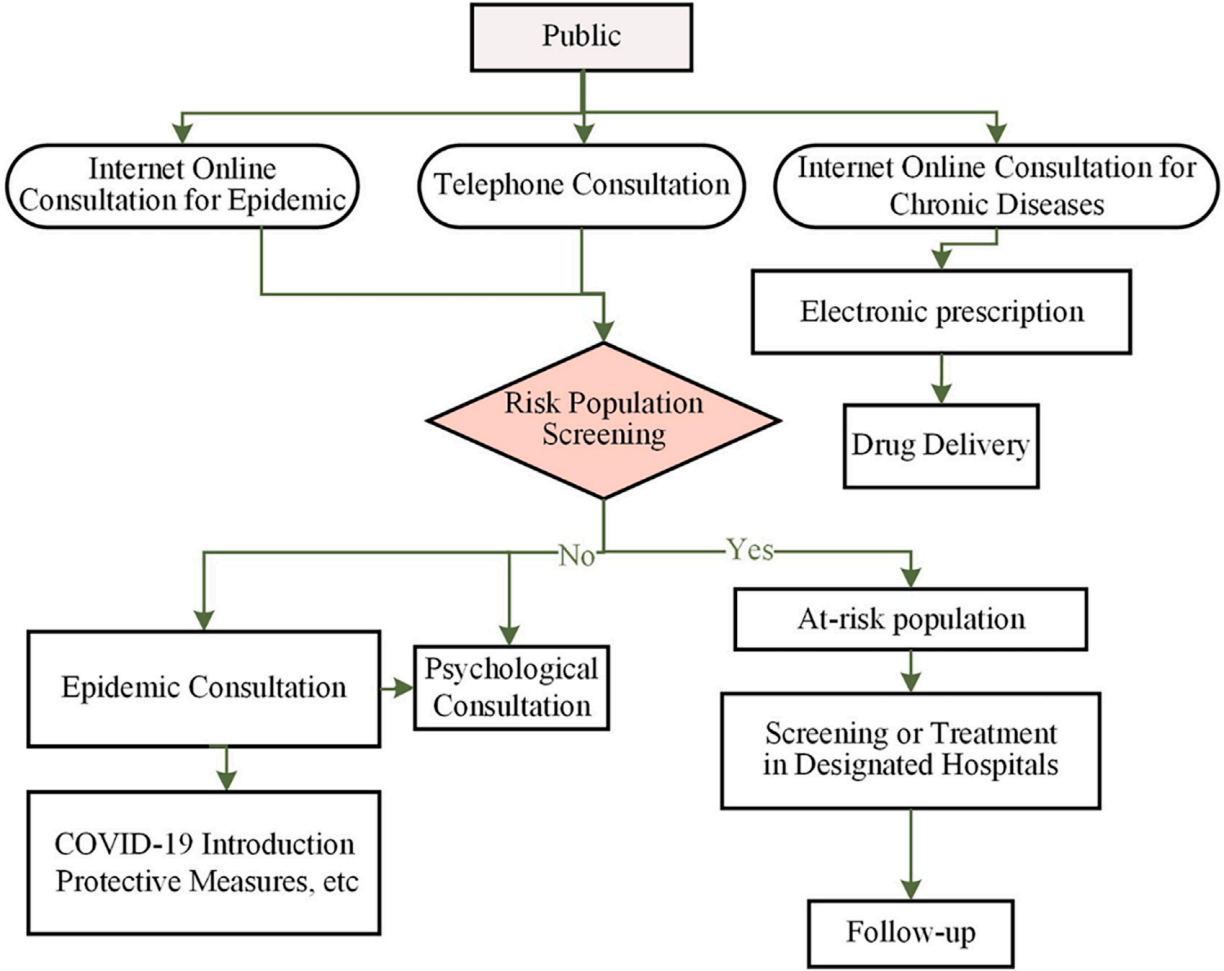
The work flow of online mode for epidemic management. The on-line project working team is divided into four groups, administrative group, Information group, Consultation group and Follow-up group.

The online and telephone prevention and control plan was based on the internet hospital platform, which involved the following tasks: 1) Dynamic online training for healthcare personnel; 2) Scientific education on infectious diseases and online consultations; 3) Screening of high-risk populations; 4) Online consultations, prescriptions, and home delivery of medications for chronic disease patients. The hospital’s IT department issued CA certificates to all doctors participating in online consultations and provided training on online operations. The pharmacist team reviewed the online electronic prescription process. A logistics company was responsible for medication delivery, and the medical internet service team handled medication consultations. The overall process of the hospital’s online model is shown in the diagram (Figure 1). During this process, some patients avoided hospital visits, thus reducing the likelihood of exposure.

### Online consultations via internet hospital during the epidemic

Through online consultations provided by the internet hospital, high-risk individuals were screened based on the diagnostic guidelines issued by the National Health Commission of China. The services included scientific education, epidemic-related consultations, and psychological services. The online screening of the population could be conducted using AI-assisted self-assessment, utilizing an information collection system where patients could autonomously gather symptom data through multimodal inputs, including text, voice, and images. Patients described their symptoms and experiences, and the system automatically analyzed the uploaded data to rank suspected cases, generating a list of suspected patients based on confidence levels [9–11]. If necessary, individuals with suspected cases were advised to visit designated hospitals for diagnosis or follow-up treatment.

**FIGURE 2:**
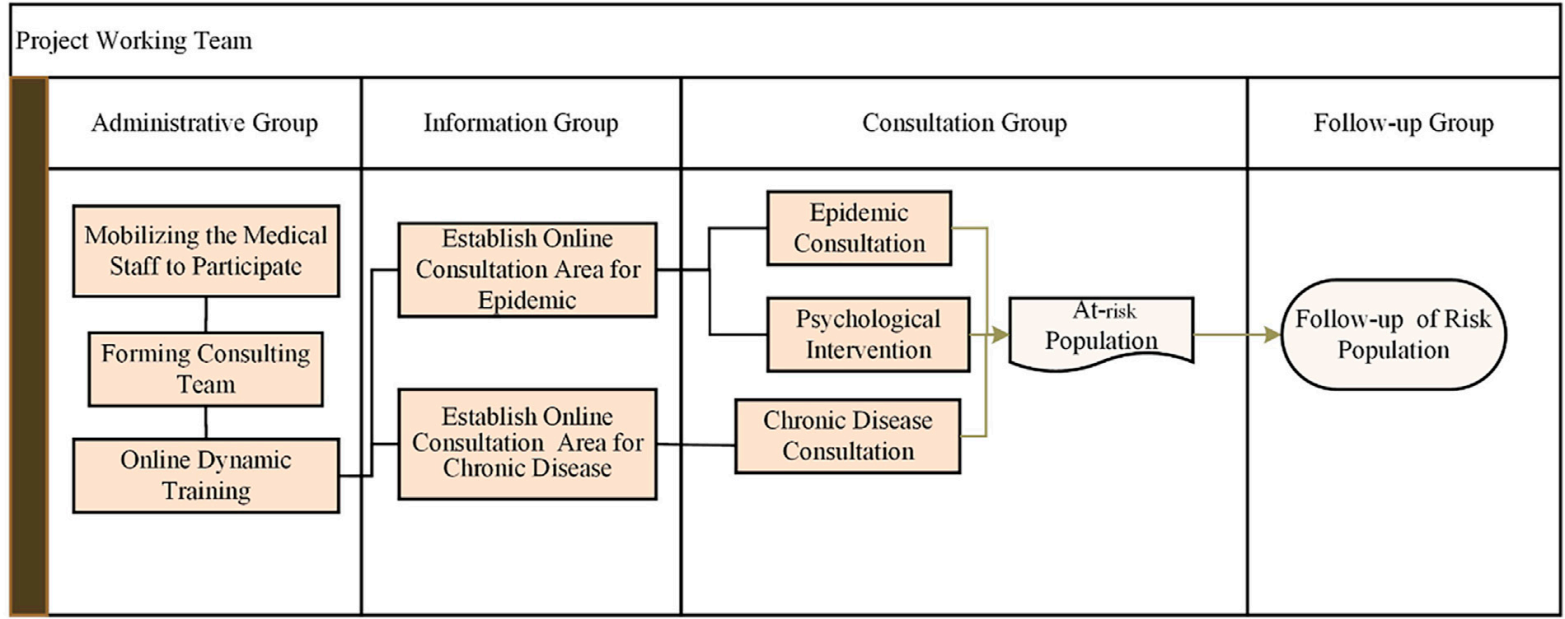
Online model consultation operation process. The on-line consultation includes two main activities, consultation for epidemic and consultation for chronic diseases. Scientific education, psychological consultation, and chronic diseases prescriptions are achieved online, meanwhile the persons at risk are screened.

A knowledge base, containing professional medical guidelines and research content, was constructed to store various disease-related information and support scientific education. A team consisting of medical experts, IT specialists, and data managers was established to update, review, and maintain the Clinical Decision Support System (CDSS) knowledge base. Regular updates ensured that the latest clinical research findings and guidelines were promptly integrated into the system. The scientific education provided included introductions to infectious diseases, protective measures, mask-wearing methods, disinfection, and handwashing techniques. For those experiencing anxiety and stress due to the epidemic, a team of professional psychologists offered psychological counseling services. The process of online consultation during the epidemic is illustrated in a diagram (Figure 2). For patients with a clear epidemiological history, we provided one-on-one guidance to direct them to the nearest location for nucleic acid testing and quarantine patients with positive testing results.

#### Pre-consultation medical history collection

Patients were free to describe their symptoms and sensations using voice, text, or a combination of both. The system analyzed the patient’s condition and automatically provided a ranked list of suspected diseases along with confidence levels. Patients could also choose to further specify their symptoms with additional options, such as selecting the affected body part, to describe their condition in more detail, thereby receiving more accurate department recommendations. Based on these recommendations, patients could choose to continue with either online or in-person consultations until the consultation was completed.

#### Knowledge base construction

The intelligent triage system required a knowledge base to store information about hospital departments, doctors’ availability for online and offline consultations, disease manifestations, and disease names. In this study, the disease knowledge base was derived from professional medical guidelines and textbooks, integrated with a large number of outpatient medical records, and confirmed by authoritative experts in the field.

### Online consultations and medication delivery for chronic disease patients

Chronic disease patients are those who require long-term medication and regular hospital visits for prescription refills. To avoid cross-infection during the epidemic, chronic disease patients described their recent health status to doctors via the online consultation platform. Each department established its own project team and developed corresponding follow-up procedures. A multidisciplinary team (MDT) outpatient clinic was set up as a central hub for chronic disease patients. The system pre-sorted the patient’s condition based on pre-visit online data collection and consultation content. For patients with unclear diagnoses, the MDT treatment team jointly discussed and made decisions, categorizing chronic disease patients and directing them to the appropriate specialized department, thereby improving diagnostic efficiency. If the patient’s condition was stable and showed no significant changes, the doctor would send an electronic prescription to the pharmacy department. After the pharmacist approved the prescription, the patient could pay online, and the delivery company would deliver the medication to the patient’s home. During the epidemic, registration fees were waived. According to the department’s follow-up procedures, doctors could track the patient’s condition and make adjustment to treatment and medication as needed at various follow-up points [12].

#### Departmental project teams

Each department established its own project team as needed and assigned team members with specific roles (administrator, disease manager, and management assistant). The follow-up process for each disease was then developed, with each follow-up process containing multiple follow-up points, which were visually displayed using a timeline to show the patient’s status changes after enrollment.

#### MDT outpatient clinic

Inter-hospital MDT patients often require comprehensive treatment involving multiple disciplines. To facilitate this, our hospital established a green channel for post-consultation MDT patients, allowing appointments for follow-up consultations to be made during the consultation, or transferring the patient to the relevant specialist within the team with a single click. This helps patients receive timely consultations and enables the team of doctors to follow up on the patient’s condition and adjust medication plans as needed. If the patient requires hospitalization, MDT office staff can stamp the admission certificate with a major disease green channel seal, helping patients gain quick admission and thereby improving patient satisfaction.

### Telephone consultations

Since the first recorded medical telephone consultation in 1879, telephone consultations have become a key component of modern patient-centered healthcare systems. Today, more than a quarter of nursing consultations are conducted via telephone. To ensure that people of all ages could participate in epidemic consultations, we established telephone consultation services. Each department utilized machine learning to analyze large amounts of patient data and consultation records, predicting potential trends in issues or changes in user needs, and making timely adjustments to service strategies while preparing the necessary medical resources in advance. Considering that some elderly people might not be familiar with smartphones or the internet, they could call the hospital hotline, and the system would transfer their calls to the mobile phones of doctors in the epidemic consultation group. These consultations included high-risk population screening, scientific education, epidemic situation updates, and psychological counseling.

## Results

**FIGURE 3:**
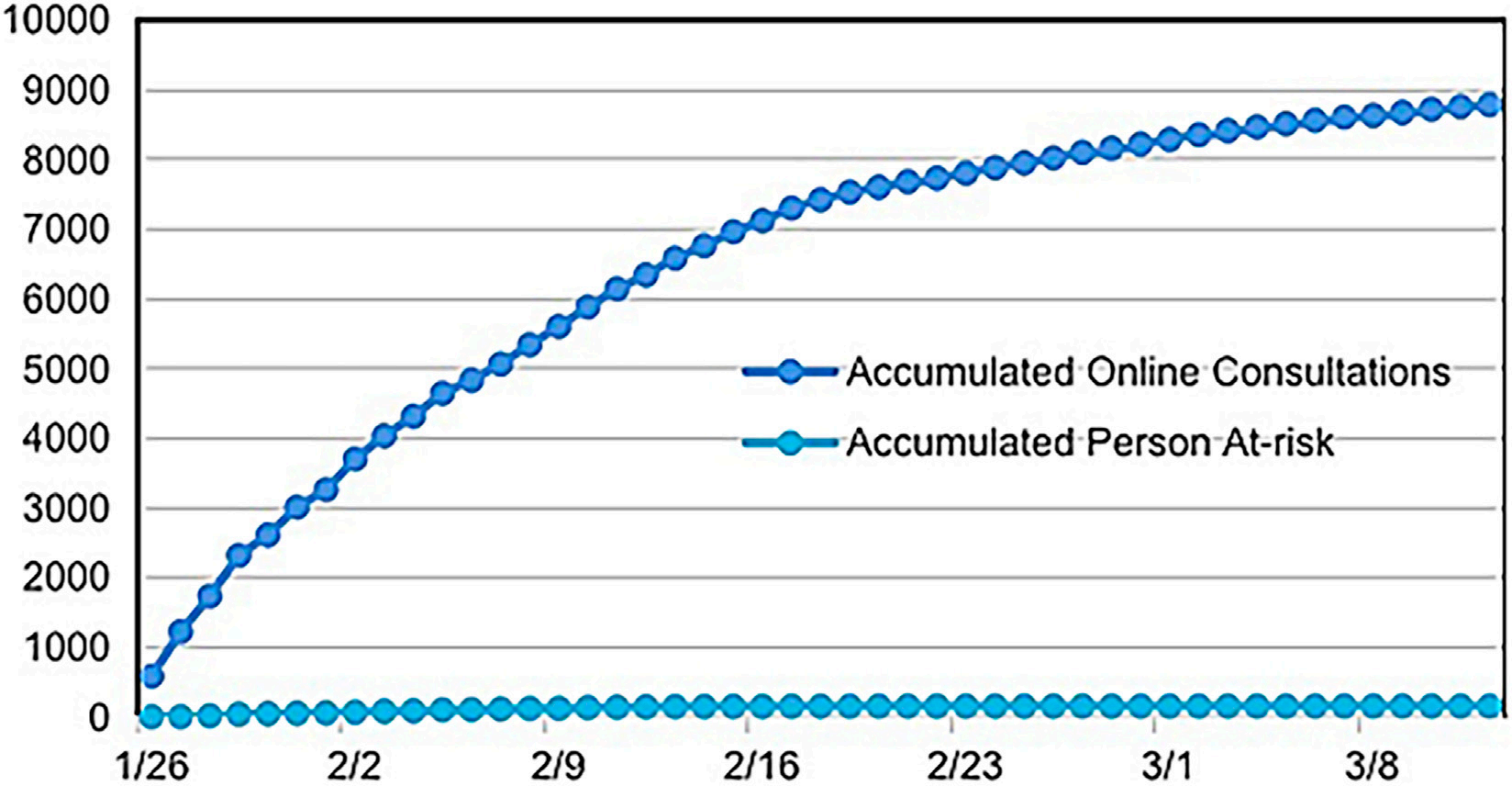
Data of internet online consultation for population in the epidemic from 29 January 2020, to 12 March 2020.

As of 12 March 2020, a total of 485 healthcare workers had participated in online training and engaged in online consultations and follow-up work. Sixteen training materials were utilized, covering information about COVID-19, including the “Diagnosis and Treatment Protocol for Novel Coronavirus Pneumonia” issued by the National Health Commission of China (currently updated to the seventh edition) and recommendations from the Chinese Center for Disease Control and Prevention. Training sessions were provided once daily, allowing healthcare workers to learn online. Criteria for identifying potential suspected COVID-19 cases were established based on individual epidemiological histories and clinical symptoms. In summary, the screening process involved asking the following questions: 1) Whether the individual had lived in or traveled to Hubei Province within 14 days prior to the consultation; 2) Whether the individual had been in contact with a COVID-19 patient reported in the epidemic reporting internet system in the past 14 days; 3) Whether the individual had been in contact with someone from Hubei Province who had a fever or difficulty breathing in the past 14 days; 4) Whether the individual had a fever or difficulty breathing; 5) Whether the individual had CT imaging similar to COVID-19 infection; 6) Whether the individual had lymphopenia. As of 12 March 2020, 205 doctors conducted 8,783 online consultations through the epidemic internet platform, averaging 187 online consultations per day, and identified 154 potential high-risk suspected cases (Figure 3).

**FIGURE 4:**
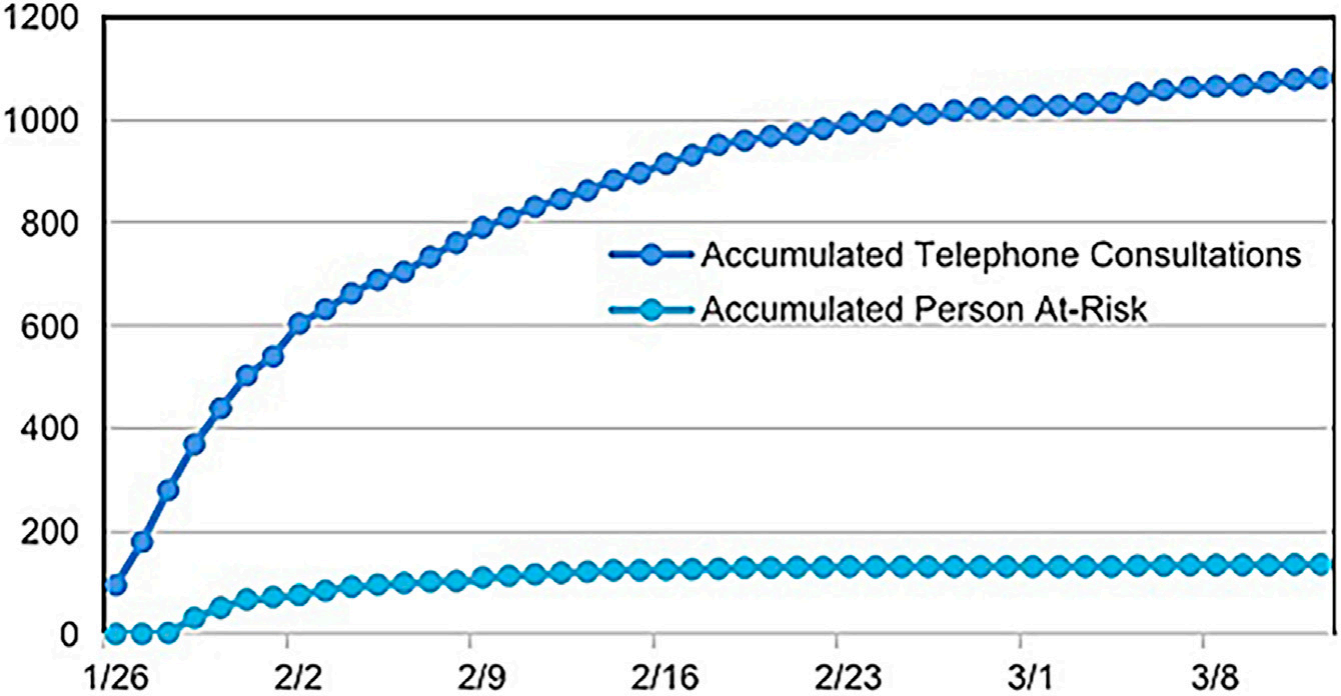
Data of telephone consultation for population in the epidemic from 29 January 2020, to 12 March 2020.

Telephone consultations began on 29 January 2020. As of 12 March 2020, a total of 1,082 telephone consultations had been conducted, with an average of 23 calls per day, identifying 135 potential high-risk suspected COVID-19 cases. As of 12 March 2020, a total of 289 potential COVID-19 risk patients were identified through online and telephone consultations, including one patient who had been diagnosed at the hospital but participated in online consultations. All high-risk patients were followed up daily, and they were guided to visit designated hospitals for further screening or diagnosis. During the follow-up period, three suspected COVID-19 patients were diagnosed and treated at designated hospitals. The four confirmed patients were in stable condition. No infections were detected in the other patients during follow-up. (Figure 4).

**FIGURE 5:**
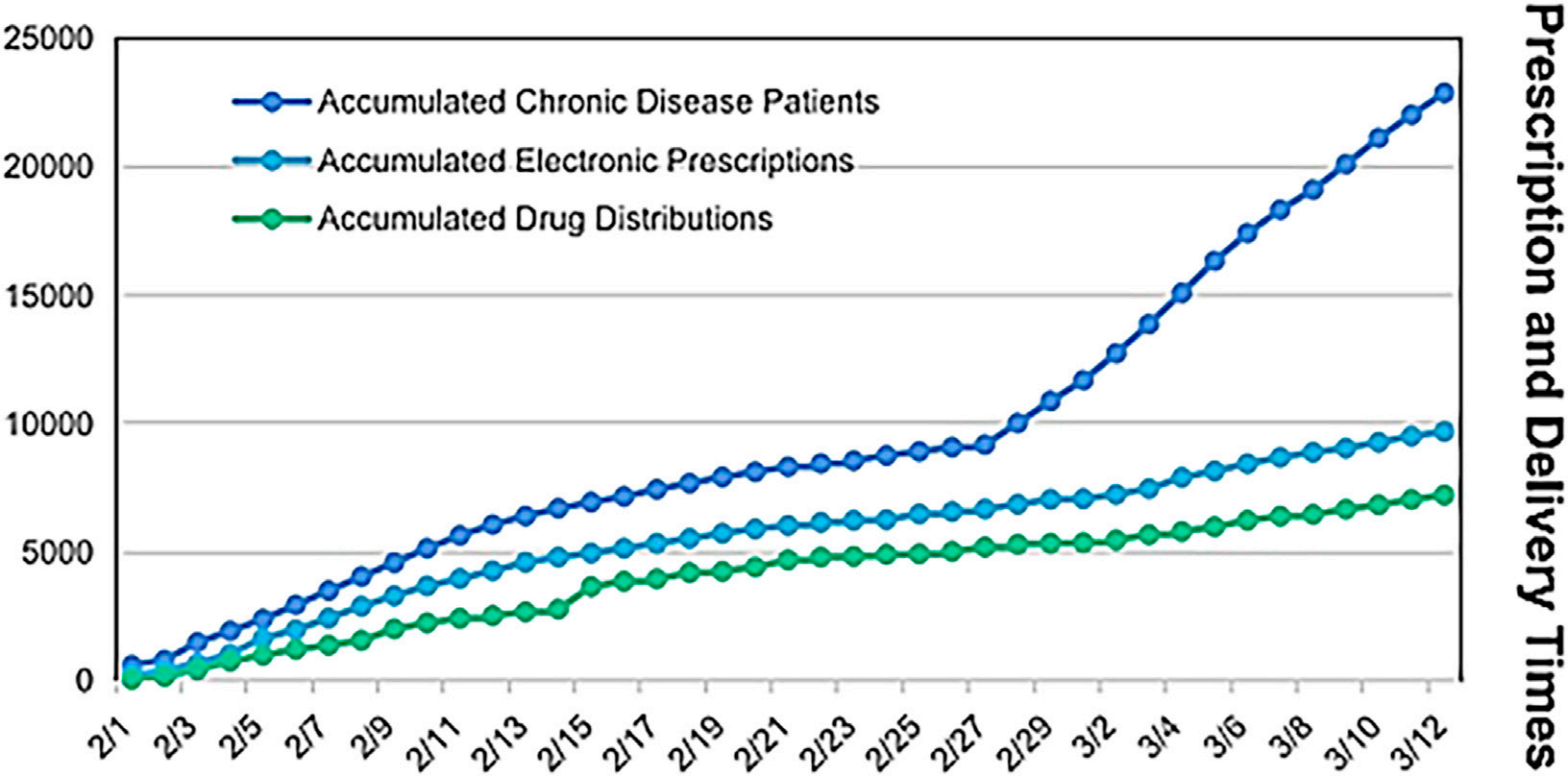
Online consultation, prescriptions and medicine distributions for chronic patients in the epidemic from 29 January 2020, to 12 March 2020.

As of 12 March 2020, 22,890 online consultations for chronic disease patients had been conducted, resulting in 9,690 electronic prescriptions and 7,218 medication delivery orders. The average daily consultation volume for chronic disease patients was 558 cases, with an average of 236 valid electronic prescriptions and 176 medication deliveries per day. During the epidemic, chronic disease patients were exempted from registration fees and delivery charges through the online consultation and medication delivery platform (Figure 5).

## Discussion

We have reported a pioneering case of online medical strategies and outcomes during an infectious disease epidemic. Infectious diseases like COVID-19 primarily spread through human-to-human transmission in public spaces and during hospital visits, similar in some ways to severe acute respiratory syndrome coronavirus (SARS-CoV). Potentially infected individuals (i.e., asymptomatic carriers) can also become sources of transmission [8, 13–15]. Patients may not have a fever or obvious symptoms but can still spread the virus to others [16]. Respiratory droplets and contact transmission are the main routes of spread. Individuals with weaker immune systems, such as the elderly, are more susceptible to COVID-19, and these elderly individuals often suffer from various chronic diseases. For highly contagious viruses like COVID-19, which are difficult to control, reducing contact and gatherings among people is an effective way to control the spread. The online consultation and diagnosis model aligns with the principle of reducing population movement and has proven to be an effective method for preventing and controlling infectious diseases. Online psychological consultations for the public provided a safe channel to reduce their panic, despair, and depressive symptoms during the COVID-19 outbreak.

The advantages of the online model include: 1) No need for in-person hospital visits, which reduces hospital traffic and lowers the risk of cross-infection between patients and between patients and healthcare workers; 2) No restrictions of time and space. People can consult doctors anytime and anywhere, without being limited by geographical location and time constraints. Doctors can complete online consultations and provide advice during working hours or even after hours; 3) Online follow-up for chronic diseases. Patients with chronic illnesses can consult professional doctors from home. Doctors issue electronic prescriptions, and medications are delivered to the patient’s home, breaking the limitations of traditional healthcare, especially suitable during an epidemic. Over approximately 1 month, the online hospital model facilitated 32,755 online interactions, including 8,783 online consultations, 1,082 telephone consultations, and 22,890 follow-ups for chronic disease patients. The online model enabled rapid screening of high-risk populations, and suspected cases were further diagnosed and treated at designated hospitals. Through online consultations, people were informed about preventive measures such as proper mask-wearing, disinfection, frequent handwashing, reducing outings, and avoiding large gatherings. This model is not only effective for densely populated countries but also invaluable for countries with limited medical resources [17]. However, the online screening protocols evolved gradually, and not all participating clinicians acquired the necessary knowledge in a short period. At the same time, inconsistencies in screening procedures may have occurred, making it challenging to ensure accurate and sensitive screening uniformly.

The internet-based online model provided electronic prescriptions and medication delivery services for chronic disease patients. Most chronic disease patients, such as those with diabetes, cancer, or liver transplants, tend to be relatively stable over short periods. Many patients were able to efficiently communicate with specialized doctors through the online platform. Doctors could assess whether the patient’s condition had significantly changed and determine whether the previous treatment plan should continue. If the condition remained stable, medications were delivered directly to the patient’s home via electronic prescriptions. This online platform brought significant convenience to chronic disease consultations, electronic prescriptions, and medication delivery, while reducing the risk of cross-infection from contact with others at the hospital. By leveraging this internet-based online model during the epidemic, hospital pressure was alleviated, and it contributed to the broader effort in controlling the epidemic.

During infectious disease outbreaks, the increased tension and anxiety among the public further complicate epidemic prevention and control efforts. Online psychological counseling has been shown to alleviate public tension and anxiety [17]. Public emergencies, especially epidemics like COVID-19, often trigger emotional responses under emergency conditions. Many people experience tension and anxiety, with some even panicking. The public frequently worries about whether they have come into contact with infected individuals or whether they themselves are at risk of infection. They experience tension, fear, anxiety, and even become suspicious of everyone around them, harboring negative and pessimistic emotions toward others. If the public’s tension and anxiety are not addressed promptly, it may lead to widespread panic. These behaviors can be assessed through online evaluation tools on the platform. After online consultations, as people gain knowledge about the epidemic and understand their own circumstances, their tension and anxiety are significantly reduced.

A robust information system is crucial for epidemic prevention and control in an internet-based online model [18]. Such a system must be supported by healthcare institutions with strong medical backing and a comprehensive information platform. The development of internet hospitals has met the needs of epidemic prevention. To ensure the smooth implementation of the online model, an “Online Project Task Force for COVID-19 Epidemic” was urgently established. Before the outbreak, the IT department had already developed an internet hospital platform, including applications for use by both doctors and the public. After the outbreak, the IT department developed online channels for epidemic consultations and chronic disease follow-ups, providing an internet platform for epidemic-related consultations, psychological counseling, and chronic disease management. The platform also allows for the issuance of electronic prescriptions. After patients make online payments through social insurance, medications are delivered to their homes by courier services.

In some infectious disease outbreaks, the rapid development of online models based on internet hospitals may lead to less user-friendly experiences and insufficient levels of intelligent interaction. The overall process can be quite complex and requires further optimization and improvement. With the continuous advancement of artificial intelligence methods, big data, and 5G technology [19], an “intelligent decision-based online model” (further discussion) could better support epidemic prevention and control in the future.

To increase the level of intelligence, we considered using artificial intelligence methods for further improvement work as follows.

1) Risk Warning Module in Telephone Consultation System: A risk warning module could be constructed within the telephone consultation system. Potential risk factors, such as fraudulent calls or malicious complaints, might arise during telephone consultations. A federated learning model could be trained to identify these potential risks and take corresponding preventive measures. Since participants share model updates rather than raw data, this approach can effectively provide risk warnings while protecting user privacy.
2) System Interface Optimization and Customization for Departments: The user interface and operational processes of the Clinical Decision Support System (CDSS) should be continuously optimized based on feedback and needs from clinicians. Personalized customization services should be offered to ensure that the CDSS aligns more closely with the working habits of different departments and doctors.
3) Development of an Intelligent Drug Management System: An intelligent drug management system could be developed to improve the efficiency of medication management and dispensing within the pharmacy department. The system would categorize drugs and establish different inventory management strategies based on the importance and usage frequency of each drug. By analyzing historical data and forecasting demand, a scientific and reasonable procurement plan and replenishment strategy could be formulated. Real-time monitoring and automatic inventory checks would enhance the efficiency of drug management. Machine learning algorithms could be employed to analyze drug usage data, establish a risk warning mechanism, and provide real-time alerts for abnormal medication usage or potential drug interactions.
4) Development of an Intelligent Medical Assistant Based on a Knowledge Base: An intelligent medical assistant could be developed for use in smart triage, patient classification, and outpatient guidance. Departments could jointly train an intelligent medical assistant using federated learning technology, integrating a chatbot connected to the established knowledge base. This assistant would automate responses to common patient queries and consultation requests, improving the efficiency and coverage of hospital services. On the doctor’s side, the intelligent medical assistant could interconnect with other medical information systems to gather necessary diagnostic data, provide relevant disease knowledge, and recommend personalized medical plans as diagnostic references, helping to address the issue of insufficient knowledge among some clinicians in a short time. Leveraging big data, the intelligent medical assistant could utilize machine learning to analyze large datasets of patient information and consultation records, predict potential trends in issues or changes in user needs, and update models at local levels using their patient data, with updates then aggregated and distributed back to the assistant centers to timely adjust service strategies and prepare the necessary medical resources.

In recent years, online platforms based on internet hospitals have developed rapidly for the prevention and control of infectious diseases. These online platforms typically offer functions such as online consultations, remote diagnostics, health monitoring, and case management Our research provided a remote, contactless hospital online model that provided the public with online medical consultations, professional psychological counseling, and chronic disease management consultations, significantly enhancing the efficiency and precision of infectious disease prevention and control.

## Data Availability

The original contributions presented in the study are included in the article/supplementary material, further inquiries can be directed to the corresponding author.
